# Adverse events of neoadjuvant combination immunotherapy for resectable cancer patients: a systematic review and meta-analysis

**DOI:** 10.3389/fimmu.2023.1269067

**Published:** 2024-01-05

**Authors:** Yuqian Feng, Kaibo Guo, Huimin Jin, Jing Jiang, Menglei Wang, Shengyou Lin

**Affiliations:** ^1^ Hangzhou School of Clinical Medicine, Zhejiang Chinese Medical University, Hangzhou, Zhejiang, China; ^2^ Department of Oncology, Hangzhou First People’s Hospital, Hangzhou, Zhejiang, China; ^3^ Department of Oncology, The Second Affiliated Hospital of Zhejiang Chinese Medical University, Hangzhou, Zhejiang, China; ^4^ The Third School of Clinical Medicine, Zhejiang Chinese Medical University, Hangzhou, Zhejiang, China; ^5^ Department of Oncology, The First Affiliated Hospital of Zhejiang Chinese Medical University (Zhejiang Provincial Hospital of Chinese Medicine), Hangzhou, Zhejiang, China

**Keywords:** neoadjuvant immunotherapy, immune checkpoint inhibitors, adverse events, safety, resectable cancer

## Abstract

**Background:**

Neoadjuvant combination immunotherapy is changing the treatment landscape for patients with cancer. Exploring the incidence of immune-related adverse events (irAEs) in relation to this novel approach may provide valuable insights for future clinical investigations.

**Methods:**

This review was conducted in accordance with the Preferred Reporting Items for Systematic Reviews and Meta-Analyses (PRISMA) guidelines. PubMed, Embase, Cochrane Library, American Society of Clinical Oncology (ASCO), and European Society of Medical Oncology (ESMO) websites were searched for all relevant literature from their inception to November 24, 2023. We then extracted the required data from the included studies and used the R software to analyze the pooled incidence of irAEs. Subgroup analyses examined the pooled incidence of irAEs according to cancer and combination types using a random-effects model.

**Results:**

Sixteen studies involving 501 patients were included in the meta-analysis. Considering the heterogeneity of the study design, we analyzed the randomized controlled studies (RCTs) and the single-arm studies separately. In RCTs, the incidence of any-grade irAEs was 95.0% (95% confidence interval [CI] 87.3-99.3) and that of grade ≥3 irAEs was 24.0% (95% CI 13.7-36.0). In single-arm studies, the incidence of any-grade irAEs was 89.4% (95% CI 75.0-98.0) and grade ≥3 irAEs was 20.3% (95% CI 8.7-35.2). In both RCTs and single arms, the most common any- grade irAEs were rash and fatigue, while the most common grade ≥3 irAEs was abnormal liver function and colitis. Due to irAEs, 9.4% of patients in RCTs and 6.9% of patients in single-arm studies did not complete the prescribed neoadjuvant treatment cycle.

**Conclusion:**

This study comprehensively summarized the incidence of irAEs in neoadjuvant combination immunotherapy. The occurrence of irAEs varies depending on the cancer and combination types. Our meta-analysis provides clinicians with essential guidance for the management of patients with cancer.

**Systematic review registration:**

https://www.crd.york.ac.uk/prospero, identifier CRD42023387969.

## 1 Introduction

In recent years, immune checkpoint inhibitors (ICIs), such as programmed death-1 (PD-1), programmed death ligand-1 (PD-L1), and cytotoxic T-lymphocyte antigen-4 (CTLA-4), have made significant progress in the treatment of various tumors (1, 2). Compared with conventional chemotherapy, ICIs can significantly extend the overall survival time of cancer patients and reduce the occurrence of adverse events (AEs) (3, 4). Currently, ICI neoadjuvant therapy is used for various tumors, such as lung cancer (5), melanoma (6), and esophageal cancer (7). In a meta-analysis of NSCLC, neoadjuvant immunotherapy was associated with significantly higher rates of pathological complete responses than neoadjuvant chemotherapy (8). Owing to its remarkable therapeutic effects, a large number of clinical trials have been conducted to investigate the efficacy and safety of neoadjuvant immunotherapy for malignant tumors.

The combination of the two ICIs has become an essential component of neoadjuvant immunotherapy. In a phase II clinical study, patients with resectable malignant melanoma who received neoadjuvant combination immunotherapy achieved a pathologic complete response rate of 57% (9). The NICHE-2 study showed that neoadjuvant combination immunotherapy in patients with colon cancer resulted in a high rate of pathologic complete response (10). In addition to the effectiveness of neoadjuvant immunotherapy, safety is a concern, especially for dual-agent immunotherapy. Most immune-related adverse events (irAEs) tend to be self-limiting or ameliorated using multiple strategies. However, in some cases, life-threatening fatal events can occur (11).

Considering that a new approach for combination immunotherapy may be added to neoadjuvant therapy for malignant tumors in the near future, it is necessary for clinicians to gain a comprehensive understanding of the safety of this approach. In this review, we conducted a comprehensive safety assessment by searching for clinical trials of neoadjuvant combination immunotherapy for malignancies and pooled the incidence of irAEs by meta-analysis.

## 2 Methods

This study was performed according to the Preferred Reporting Items for Systematic Reviews and Meta-analyses (PRISMA) statement (12) and was registered at PROSPERO (CRD42023387969).

### 2.1 Literature search and screening

Three databases PubMed, Embase, Cochrane Library, American Society of Clinical Oncology (ASCO), and European Society of Medical Oncology (ESMO) websites were searched for relevant literature from their inception until November 24, 2023. The search strategy for PubMed is presented in **Supplementary Table S1**. After importing documents into Endnote reference management software (Version X9), filtering was performed.

According to the population, issue of interest, comparison, outcome, and study design (PICOS) method (13), studies meeting the following criteria were included: (i) populations: the study was conducted in resectable cancer patients, (ii) interventions: neoadjuvant combination immunotherapy was used as an intervention (only dual immune checkpoint inhibitors were included), (iii) outcomes: irAEs for outcome indicators were reported, (iv) study designs: RCTs, single-arm, and non-RCT studies were all included, and (v) the full text was published in English. The exclusion criteria were as follows: (i) neoadjuvant immunotherapy combined with other treatment modalities, such as chemotherapy, targeted therapy, and radiotherapy, (ii) no mention of irAEs in the outcome indicator; and (iii) only a summary without a full-text report. The above processes were independently completed by two authors.

### 2.2 Data extraction and quality assessment

Two authors carefully reviewed the eligible literature and extracted relevant information, including the first author, publication year, study design, cancer type, intervention, number of patients, surgical time, follow-up time, case of irAEs (any grade and high grade), completion of neoadjuvant therapy, case of surgery delayed or not performed, and evaluation criteria of AEs.

The quality of randomized controlled trials (RCTs) was assessed using the Cochrane Collaboration’s risk of bias tool (14). Additionally, a quality assessment of non-randomized controlled studies was performed using the methodological index for non-randomized studies (MINORS) checklist (15). Two authors independently assessed the quality of the literatures and promptly consulted a third author regarding points of dispute.

### 2.3 Statistical analysis

The primary objective of our meta-analysis was to determine the incidence of irAEs, including any-grade irAEs as well as high-grade irAEs. The pooled incidence with 95% confidence intervals (CIs) was calculated using a random effects model in RCTs and single-arm studies, respectively. Subgroup analyses were performed to examine the pooled incidence of irAEs according to cancer and combination types. Rate consolidation was conducted using arcsine transformation. Heterogeneity among the pooled studies was assessed using the heterogeneity index (I^2^).

All of the above analyses were performed using R software (version 4.1.0) with the Meta package. A *P* value of <0.05 was considered statistically significant.

## 3 Results

### 3.1 Eligible studies and characteristics

**Figure 1:**
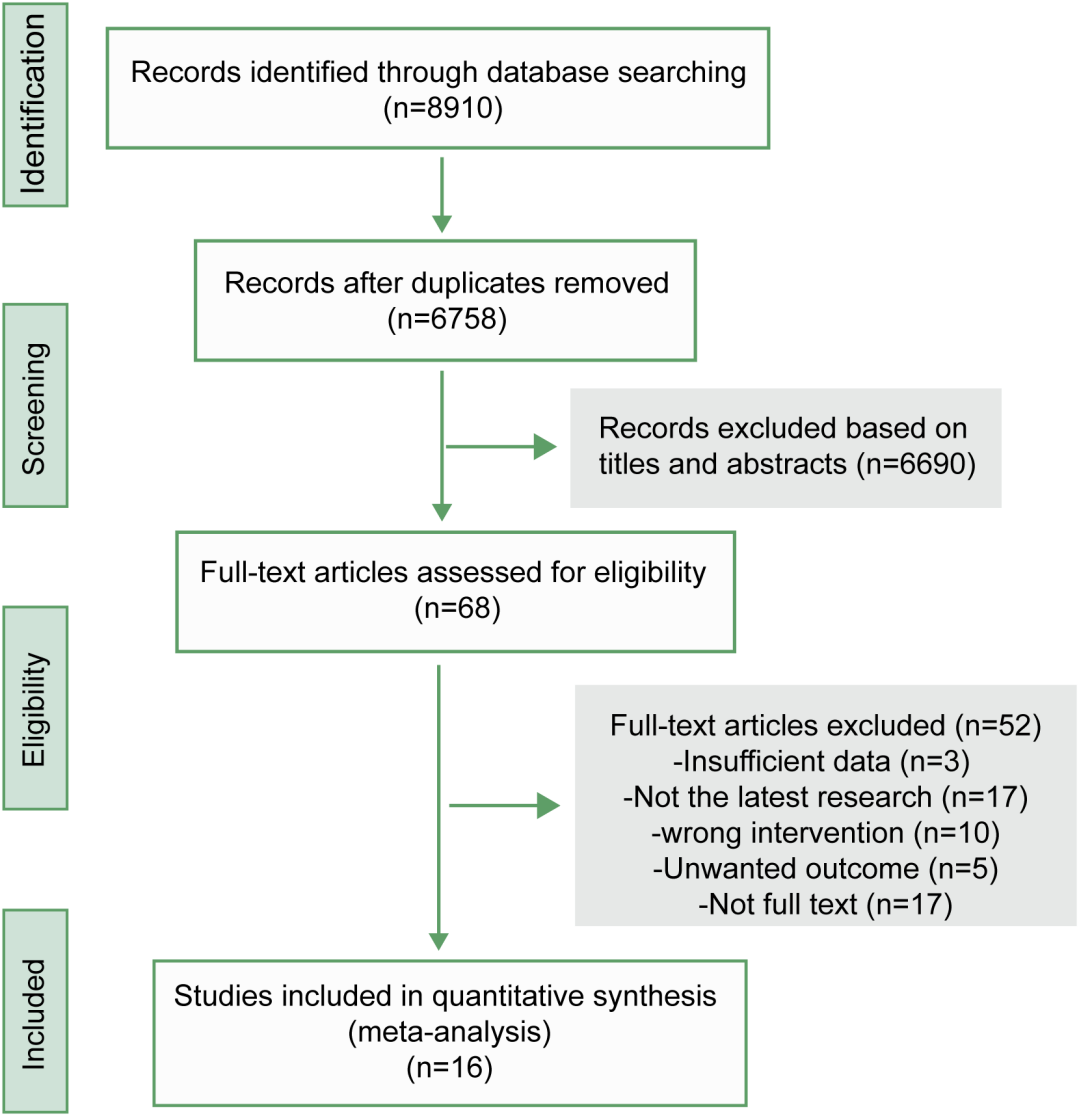
Flow chart of the literature selection process.

Through a systematic literature search, 8910 relevant records were retrieved. Duplicates were removed, and titles and abstracts were browsed, leaving 68 records that required full-text reading. Among them, 3 did not have sufficient data, 17 were not the latest study results, 10 were wrong interventions, 5 were wrong outcome indicators, and 17 were not found in the full text. Sixteen studies were included; nine were non-RCTs (including eight single-arm studies) and seven were RCTs (**Figure 1**). The risk of bias for RCTs is detailed in **Supplementary Table S2**, while that for non-RCTs is detailed in **Supplementary Table S3**.

The main characteristics of sixteen included clinical trials are summarized in **Table 1**. All publications were published after 2019. In total, 501 patients received neoadjuvant combination immunotherapy. In four studies (16–19), 76 patients received durvalumab plus tremelimuma as neoadjuvant therapy. In nine studies (20–28), 326 patients received nivolumab plus ipilimumab. Thirty patients received relatlimab plus nivolumab (9), 28 patients received lirilumab plus nivolumab (29), 21 patients received Durvalumab plus Oleclumab, and 20 patients received Durvalumab plus Monalizumab (30). AEs in thirteen studies were assessed using the National Cancer Institute’s Common Terminology Criteria, whereas those in three studies were not reported.

**Table 1:** Main characteristics of all included studies.

First author	Publish year	Study design	Cancer type	Intervention (T)	Intervention(C)	Cycle	Number of patients (T/C)	surgical time	Follow-up time	Criteria for adverse events
Schoenfeld, J.D.	2020	RCT	HNC	Nivolumab 3 mg/kg+ Ipilimumab 1 mg/kg	Nivolumab 3 mg/kg	Two cycles nivolumab	15/14	19d (7-21d), initiation of ICIs	14.2M	CTCAE v4.0
Lee, H. S.	2022	RCT	Pleural Mesothelioma	Durvalumab 1500mg+ Tremelimumab 75 mg	Durvalumab 10mg/kg	One cycle	11/9	22d (14-61d), last dose of ICIs	34.1M	NR
Ferrarotto, R.	2022	RCT	HNC	Durvalumab 1500mg+ Tremelimumab 75 mg	Durvalumab 1500mg	Two cycles	14/15	56d(52- 72d), initiation of ICIs	15.79M(8.38–23.83)	CTCAE v4.03
Cascone, T.	2022	RCT	NSCLC	Nivolumab 3mg/kg+ Ipilimumab 1mg/kg	Nivolumab 3mg	Three cycles nivolumab	21/23	31d (21-87d), last dose of ICIs	22.2M	NR
Kaseb, A.O.	2022	RCT	HCC	Nivolumab 240 mg+ Ipilimumab 1mg/kg	Nivolumab 240 mg	Three cycles nivolumab	14/13	NR	24.6M(21.1–32.9)	CTCAE
Rozeman, E. A.	2019	RCT	Melanoma	Ipilimumab 3 mg/kg+ Nivolumab 1 mg/kg; Ipilimumab 1 mg/kg +Nivolumab 3 mg/kg	Ipilimumab 3 mg/kg + Nivolumab 3 mg/kg	Two cycles	30/30/26	6w, initiation of ICIs	8.3M(5.6–11.7)	CTCAE v4.03
Cascone, T.	2023	RCT	NSCLC	Durvalumab 1500mg+Oleclumab 3000mg, Durvalumab 1500mg + Monalizumab 750mg	Durvalumab 1500mg	NR	21/20/26	29-42d	105d	NR
Vos, J. L.	2021	Non-RCT	HNC	Nivolumab 240 mg+ Ipilimumab 1mg/kg	Nivolumab 240 mg	Two cycles nivolumab	26/6	27d, initiation of ICIs	24M(21.5-not attained)	CTCAE v4.03
Gao, J.	2020	Single-arm	UC	Durvalumab 1500 mg+ Tremelimumab 75 mg	NR	Two cycles	28	NR	19.2M	CTCAE v4.03
Reijers, I.L.M.	2020	Single-arm	Melanoma	Ipilimumab 1mg/kg+ Nivolumab 3mg/kg	NR	Two cycles	99	NR	28.1M(25.0-33.8)	CTCAE v4.03
Reuss, J.E.	2020	Single-arm	NSCLC	Nivolumab 3 mg/kg+ Ipilimumab 1 mg/kg	NR	Three cycles nivolumab	9	NR	NR	CTCAE v4.0
Marie, P.K.	2021	Single-arm	CRC	Durvalumab 1500 mg+ Tremelimumab 75 mg	NR	One cycle	23	30d (17-69d)	2.3Y	CTCAE v4.03
Amaria, R.N.	2022	Single-arm	Melanoma	Relatlimab 160 mg+ Nivolumab 480 mg	NR	NR	30	Week 9	24.4M(7.1-34.6)	CTCAE v4.03
Andre, T.	2022	Single-arm	Oeso-gastric adenocarcinoma	Nivolumab 240 mg+ Ipilimumab 1 mg/kg	NR	Six cycles nivolumab	32	35d(28-168d), last dose of ICIs	14.9M(10.6-17.6)	CTCAE v5.0
Hanna, G.J.	2022	Single-arm	HNC	Nivolumab 240 mg+ Lirilumab 240 mg	NR	NR	28	13d (6-24d)	22.8M(9.2-35.7)	CTCAE v4.0
van Dijk, N.	2020	Single-arm	UC	Ipilimumab 3 mg/kg (day 1/22) + nivolumab 1 mg/kg (day 22) + nivolumab 3 mg/kg (day 43)	NR	NR	24	Week 12	8.3M(4.7-13.1)	CTCAE v4.0

NSCLC, Non Small Cell Lung Cancer; HNC, Head and Neck Cancer; HCC, Hepatocellular Carcinoma; CRC, Colorectal Cancer; UC, Urothelial Cancer; RCT, Randomized Controlled Trial; ICIs, Immune checkpoint inhibitors; NR, Not Reported; Y, Year; M, Month; d: Day; CTCAE, Common Terminology Criteria for Adverse Events; T, Treatment arm; C, Control arm.

### 3.2 Incidence of overall irAEs

**Figure 2:**
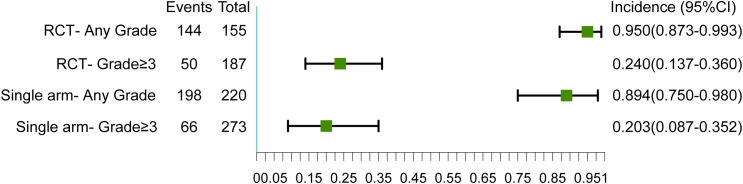
Overall incidences of any grade and grade ≥ 3 immune-related adverse events (irAEs).

In general, 15 trials reported the occurrence of grade ≥3 irAEs, with 11 reporting any-grade irAEs. Considering the heterogeneity of the study designs, we analyzed the RCTs and single-arm studies separately. The study published by Vos et al. was not included in the subsequent meta-analysis because it was neither an RCT study nor a single-arm study (20). In RCTs, the incidence of any-grade irAEs was 95.0% (95% CI 87.3-99.3) and that of grade ≥3 irAEs was 24.0% (95% CI 13.7-36.0).In single-arm studies, the incidence of any-grade irAEs was 89.4% (95% CI 75.0-98.0) and that of grade ≥3 irAEs was 20.3% (95% CI 8.7-35.2) (**Figure 2**). In addition, we measured the incidence of irAEs with immunotherapy alone. The incidence of any-grade irAEs was 87.1% (95% CI 55.9-100.0) and that of grade ≥3 irAEs was 17.3% (95% CI 10.1-25.9) (**Supplementary Figure S1**). There was no statistical difference in the incidence of irAEs between monotherapy and combination therapy (**Supplementary Figure S2**).

Considering the presence of heterogeneity, we performed a subgroup analysis according to combination and cancer types (**Table 2**). In RCTs, regarding the combination type, the incidence of any-grade irAEs was 97.1% (95%CI 90.9-99.9) and that of grade ≥3 irAEs was 32.6% (95%CI 17.6-49.7) for anti PD-1 plus anti CTLA-4 (nivolumab combined with ipilimumab). The incidence of any-grade irAEs was 100.0% (95% CI 76.8-100), while that of grade ≥3 irAEs was 15.3% (95% CI 1.6-39.0) for anti PD-L1 plus anti CTLA-4 (durvalumab combined with tremelimuma). Regarding the cancer type, the incidence of any-grade irAEs was 80.2% (95%CI 64.8-92.0) for non-small cell lung cancer (NSCLC), and 98.4% (95%CI 94.3-100.1) for melanoma. The incidence of grade ≥3 irAEs was 11.2% (95%CI 4.6-20.2) for NSCLC, 7.1% (95% CI 0.2-3.4) for head and neck cancers (HNCs), and 38.4% (95%CI 22.0-56.3) for melanoma.

**Table 2:** Subgroup analysis for irAEs according to the combination type and cancer type.

		RCTs Incidence(%,95%CI)	Single arms Incidence(%,95%CI)
Combination type			
*Anti PD-1 plus CTLA-4*	Any Grade	97.1 (90.9-99.9)	91.0 (69.5-100.0)
	Grade≥3	32.6 (17.6-49.7)	32.9 (19.1-48.3)
*Anti PD-L1 plus CTLA-4*	Any Grade	100.0 (76.8-100.0)	92.9 (76.5-99.1)
	Grade≥3	15.3 (1.6-39.0)	21.6 (11.5-33.8)
Cancer type			
*NSCLC*	Any Grade	80.2 (64.8-92.0)	66.7 (29.9-92.5)
	Grade≥3	11.2 (4.6-20.2)	33.3 (7.5-70.1)
*HNC*	Any Grade	100.0 (76.8-100.0)	75.0 (55.1-89.3)
	Grade≥3	7.1 (0.2-3.4)	10.7 (2.3-28.2)
*Melanoma*	Any Grade	98.4 (94.3-100.1)	98.0 (92.9-99.8)
	Grade≥3	38.4 (22.0-56.3)	8.5 (0.0-58.2)

In single-arm studies, regarding the combination type, the incidence of any-grade irAEs was 91.0% (95%CI 69.5-100.0) and that of grade ≥3 irAEs was 32.9% (95%CI 19.1-48.3) for anti PD-1 plus anti CTLA-4. The incidence of any-grade irAEs was 92.9% (95% CI 76.5-99.1), while that of grade ≥3 irAEs was 21.6% (95% CI 11.5-33.8) for anti PD-L1 plus anti CTLA-4. Regarding the cancer type, the incidence of any-grade was 66.7% (95% CI 29.9-92.5) for NSCLC, 75.0% (95% CI 55.1-89.3) for HNCs, and 98.0% (95% CI 92.9-99.8) for melanoma. The incidence of grade ≥3 irAEs was 33.3% (95% CI 7.5-70.1) for NSCLC, 10.7% (95% CI 2.3-28.2) for HNCs, and 8.5% (95%CI 0.0-58.2) for melanoma.

### 3.3 Incidence of specific irAEs

**Figure 3:**
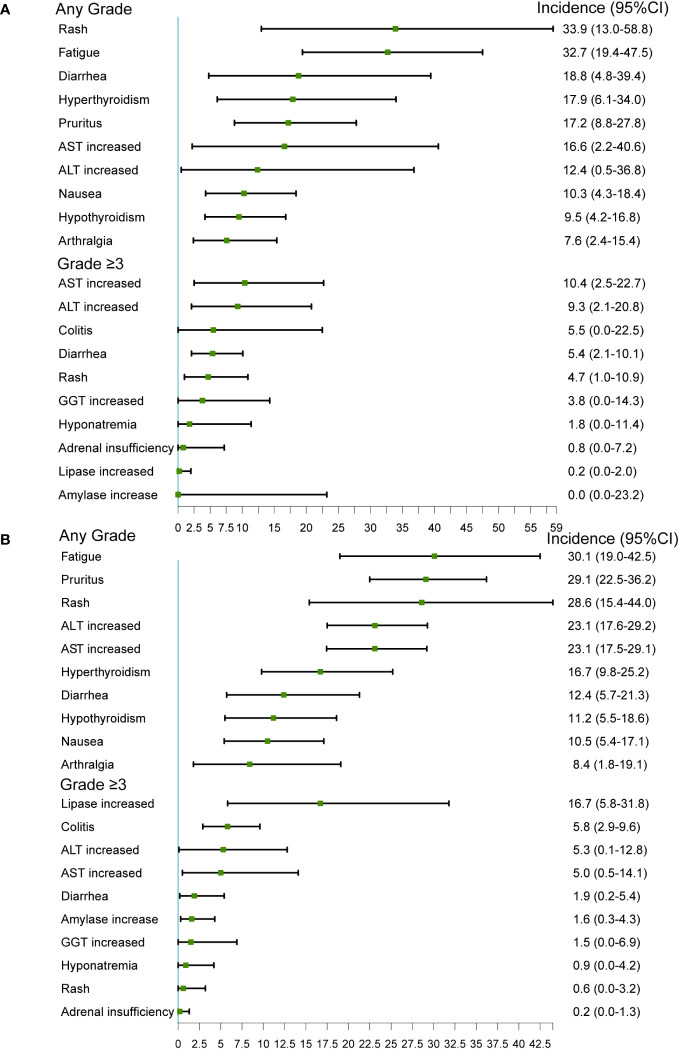
The 10 most common irAEs in RCTs and single arm studies. **(A)** RCTs; **(B)** Single-arm studies.

A total of 146 irAEs were reported in the 16 included trials; the 10 most common irAEs are described in **Figure 3**. In RCTs, the most common any-grade irAEs were rash (33.9%, 95% CI 13.0-58.8), fatigue (32.7%, 95% CI 19.4-47.5), diarrhea (18.8%, 95%CI 4.8-39.4), hyperthyroidism (17.9%, 95%CI 6.1-34.0), and pruritus (17.2%, 95% CI 8.8–27.8). The most common grade ≥3 irAEs were AST increased (10.4%, 95% CI 2.5-22.7), ALT increased (9.3%, 95% CI 2.1-20.8), colitis (5.5%, 95% CI 0.0-22.5), diarrhea (5.4%, 95% CI 2.1-10.1), and rash (4.7%, 95% CI 1.0-10.9).

In single-arm studies, the most common any-grade irAEs were fatigue (30.1%, 95% CI 19.0-42.5), pruritus (29.1%, 95% CI 22.5-36.2), rash (28.6%, 95% CI 15.4-44.0), ALT increased (23.1%, 95% CI 17.6-29.2), and AST increased (23.1%, 95% CI 17.5-29.1). The most common grade ≥3 irAEs were lipase increased (16.7%, 95% CI 5.8-31.8), colitis (5.8%, 95%CI 2.9-9.6), ALT increased (5.3%, 95% CI 0.1-12.8), AST increased (5.0%, 95% CI 0.5-14.1), and diarrhea (1.9%, 95% CI 0.2-5.4).

The most common irAEs varied among the different combination types and cancer types, as summarized in **Supplementary Tables S4–8**. In RCTs, the most common any-grade irAEs were rash (57.7%) and fatigue (42.4%) and the most common and grade ≥3 irAEs were AST increased (10.4%) and ALT increased (9.3%) in nivolumab plus ipilimumab group (**Supplementary Table S4**). The most common any-grade and grade ≥3 irAEs was hyperglycemia (54.6%, 18.2%) in durvalumab plus tremelimuma group (**Supplementary Table S5**). The most common irAEs of any-grade were acneiform rash (52.4%) in NSCLC (**Supplementary Table S6**), rash (28.6%) in patients with HNCs (**Supplementary Table S7**), and fatigue (58.2%) in melanoma group (**Supplementary Table S8**).

In single-arm studies, the most common any-grade irAEs were rash (32.0%) and fatigue (30.4%) and the most common grade ≥3 irAEs were lipase increased (14.9%) and ALT increased (8.2%) in nivolumab plus ipilimumab group (**Supplementary Table S4**). The most common any-grade irAEs was fatigue (29.6%), and the most common grade ≥3 irAEs was lipase increased (14.3%) in durvalumab plus tremelimuma group (**Supplementary Table S5**). The most common any grade irAEs were fatigue (42.9%, 35.9%) in the HNCs and melanoma groups and rash (44.4%) in NSCLC group (**Supplementary Tables S6–8**).

### 3.4 The impact of the irAEs in the treatment

**Figure 4:**
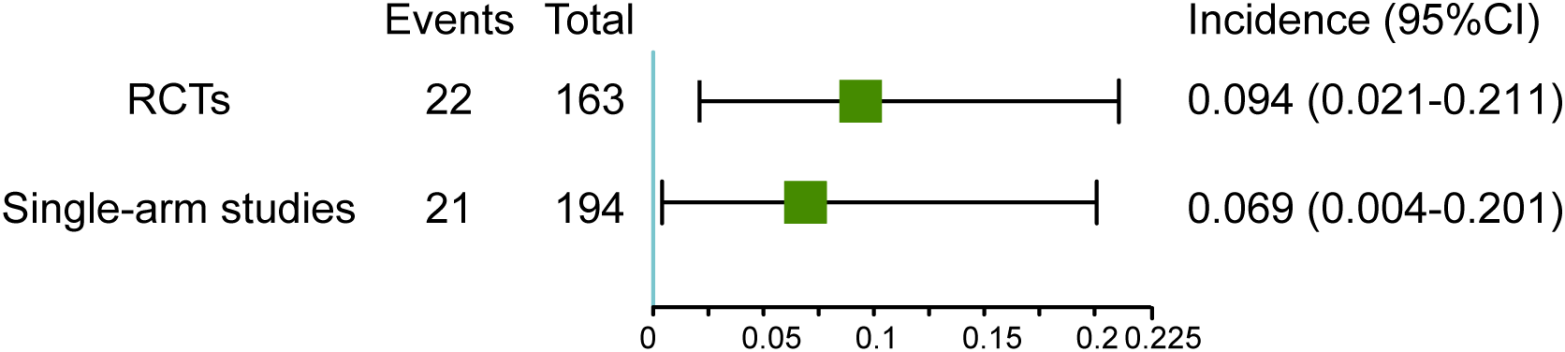
Incidence of neoadjuvant therapy discontinuation in RCTs and single-arm studies.

Due to the occurrence of irAEs, a number of patients did not complete the prescribed neoadjuvant treatment cycle. In RCTs, 9.4% (95% CI 2.1–21.1) of patients did not complete the prescribed neoadjuvant treatment cycle due to irAEs (such as grade 2-4 diarrhea/colitis, grade 2-3 ALT increased, grade 2 pneumonitis/arthralgia, grade 3 rash/meningitis/radiculitis). And in single-arm studies, 6.9% (95%CI 0.4-20.1) of patients did not complete the prescribed neoadjuvant treatment cycle due to irAEs (such as colitis/ileitis/gastritis/hepatitis) (**Figure 4**). Treatment of severe irAEs has only been reported in a few studies. Ferrarotto, R. et al. mentioned that two patients with immune-related transaminitis and diarrhea were treated with steroids (17). One patient developed pneumonia and pneumonitis requiring steroids in the research of Cascone, T (21). In addition, there has been one case of a patient with immune-related encephalitis who was treated with steroids but died (27).

A small percentage of patients have surgery delayed due to irAEs. In RCTs, only 3 of the 188 patients reported experienced surgery delayed (**Supplementary Table S9**). And in single-arm studies, only 6 of the 273 patients reported experienced surgery delayed (**Supplementary Table S10**). Eight studies reported postoperative adjuvant treatment. Most of patients chose monotherapy immunotherapy, chemotherapy or radiotherapy during the postoperative adjuvant treatment phase. In three studies reported by Amaria, R.N., Hanna, G.J., Kaseb, A.O., the adjuvant therapy was treated in the same way as the neoadjuvant phase (**Supplementary Table S11**) (9, 23, 29).

## 4 Discussion

In this meta-analysis, we found that more than 85% of patients developed irAEs during neoadjuvant immunotherapy both in the RCTs and in the single-arm studies, and approximately 1/5 of these patients developed grade ≥ 3 irAEs. Subsequently, we conducted a subgroup analysis of different cancer and intervention types. The incidence of irAEs varied among different tumors and treatment types. In addition, we found that the most common irAEs associated with neoadjuvant combination immunotherapy were fatigue, rash, abnormal liver function (ALT/AST increased), and gastrointestinal reactions (diarrhea, colitis) in both the RCTs and single-arm studies. No more than 10% of patients stopped neoadjuvant therapy and very few patients delayed surgery due to irAEs.

The administration of ICIs during neoadjuvant therapy is believed to enhance systemic T cells, which can respond to tumor antigens and activate the immune system. Compared with adjuvant therapy after surgical excision, high-burden tumors activate more circulating tumor-specific T cells, which exert beneficial anti-tumor effects (31). Liu et al. constructed a mouse model of spontaneously metastasized breast cancer that was given immunotherapy before or after surgical resection of the primary tumor, and found that the survival rate of mice receiving neoadjuvant immunotherapy was significantly higher than that of mice receiving adjuvant therapy. Improved survival was associated with more tumor-specific CD8 T cells in the blood of the mice (32). A randomized trial of recurrent glioblastoma showed a significant improvement in overall survival in the patients received neoadjuvant pembrolizumab compared to the patients recevied PD-1 blockade alone (33). Neoadjuvant therapy showed greater amplification of tumor-resident T cell clones in peripheral blood (6). However, overreactions of the immune system are also prone to irAEs, especially with combination immunotherapy (34). The incidence of severe toxicity in the neoadjuvant population appears to exceed that in the adjuvant population (31). By analyzing blood samples from patients with melanoma before ICI treatment, Lozano et al. found that activated CD4 memory T cell abundance and T cell receptor diversity were associated with severe irAEs (35). It is critical to understand the fatal events associated with ICI treatment and promote early identification, optimal assessment, and effective management. The IMCISION trial (20) showed that in patients with HNC, the use of nivolumab plus ipilimumab prior to extensive surgery was safe, but 38% of grade≥ 3 irAEs were still observed. Two ICIs increased the risk of organ-specific irAEs compared to ICI monotherapy (36). IrAEs most commonly occurred during the first 3 months of treatment but appeared at any time after treatment or even several months after treatment was terminated (37). This highlights the need for close monitoring of the occurrence of irAEs in patients during the neoadjuvant and postoperative stages.

A previous meta-analysis showed that the common AEs of adjuvant immunotherapy in patients with advanced tumors were fatigue, rash, gastrointestinal toxicity (diarrhea, nausea, and vomiting), and liver toxicity (ALT increased) (38), which was similar to our results. Once irAEs, such as immune hepatitis, gastrointestinal toxicity, occur, most can be managed by discontinue or delaying the use of ICIs or administering corticosteroids. Mild-to-moderate dermal toxicity can be effectively controlled using topical drugs; in most cases, treatment interruption is not required. Hormone replacement therapy is needed in patients with endocrine diseases, such as hypothyroidism and thyroiditis (39, 40). However, a meta-analysis has shown that corticosteroid administration is associated with poor overall survival and progression-free survival in patients with brain metastasis. Therefore, further determination of the steroid dose and duration of administration is needed to optimize survival outcomes in patients receiving ICIs (41).

Our study has several advantages. First, only AEs related to neoadjuvant immunotherapy were evaluated, which can help us understand the safety of this emerging therapy. Second, subgroup analyses based on cancer and ICI types were conducted. Third, specific AEs in neoadjuvant immunotherapy were analyzed, allowing clinicians to understand which AEs require extra attention.

However, this study had some limitations. First, the included studies were almost all single-arm, small-sample studies, and there was a large difference in sample size between individual studies. Second, the number of studies included was insufficient to analyze the safety of specific subgroups (such as the same combination of drug interventions for the same tumor type), and the presence of confounding factors may have affected the results of small-sample subgroups. Heterogeneity between studies was high, and the sources of heterogeneity could only be explored through limited subgroup analyses. Third, the occurrence, duration, and treatment of irAEs could not be analyzed. In addition, the different criteria for AEs are limitations of this study. Therefore, large-scale studies, especially RCTs, are required. We hope to provide more complete results in future studies.

## 5 Conclusion

Our study comprehensively analyzed the occurrence of irAEs in neoadjuvant immunotherapy. Most patients receiving neoadjuvant combination immunotherapy developed irAEs, and only about 20% of them developed grade ≥3 irAEs. The occurrence of irAEs varied slightly between different tumors and interventions, and mainly included cutaneous, gastrointestinal, and hepatic toxicity. Nearly 10% of patients needed to stop neoadjuvant immunotherapy due to irAEs, but few case had surgery delayed. It is important to correctly evaluate AEs and promptly intervene. An increasing number of immunotherapies are being evaluated; however, choosing the appropriate combination of drugs, order of administration, and dose to increase efficacy and reduce AEs remains a challenge.

## Data availability statement

The original contributions presented in the study are included in the article/**Supplementary Material**. Further inquiries can be directed to the corresponding author.

## Author contributions

YF: Conceptualization, Data curation, Formal analysis, Investigation, Methodology, Writing – original draft. KG: Data curation, Investigation, Methodology, Writing – original draft. HJ: Investigation, Methodology, Writing – review & editing. JJ: Investigation, Writing – review & editing. MW: Investigation, Writing – review & editing. SL: Investigation, Supervision, Validation, Writing – review & editing.
