# Supplementary material for: Adverse events of neoadjuvant combination immunotherapy for resectable cancer patients: a systematic review and meta-analysis
Source: Front Immunol. 2024 Jan 5;14:1269067. doi: 10.3389/fimmu.2023.1269067 (PMC10796654; doi:10.3389/fimmu.2023.1269067)
Supplement: Supplementary file 1 [file DataSheet_1.docx]

**Table S1 Search strategy for pubmed.**

| **#1 Tumor** | (((((("Neoplasms"[MeSH Terms]) OR (tumor*[Title/Abstract])) OR (neoplasm*[Title/Abstract])) OR (neoplasia*[Title/Abstract])) OR (cancer*[Title/Abstract])) OR (malignan*[Title/Abstract])) OR (tomour*[Title/Abstract]) |
| --- | --- |
| **#2**  **Intervetnion** | (((((((((((((((((((((((((((((((nivolumab[Title/Abstract]) OR pembrolizumab[Title/Abstract]) OR atezolizumab[Title/Abstract]) OR durvalumab[Title/Abstract]) OR cemiplimab[Title/Abstract]) OR toripalimab[Title/Abstract]) OR sintilimab[Title/Abstract]) OR avelumab[Title/Abstract]) OR Keytruda[Title/Abstract]) OR Opdivo[Title/Abstract]) OR Libtayo[Title/Abstract]) OR Tecentriq[Title/Abstract]) OR Bavencio[Title/Abstract]) OR Imfinzi[Title/Abstract]) OR Camrelizumab[Title/Abstract]) OR tremelimumab[Title/Abstract]) OR lambrolizumab[Title/Abstract]) OR MK-3475[Title/Abstract]) OR MDX-1106[Title/Abstract]) OR ONO-4538[Title/Abstract]) OR BMS-936558[Title/Abstract]) OR MPDL3280A[Title/Abstract]) OR RG7446[Title/Abstract]) OR RG-7446[Title/Abstract]) OR ipilimumab[Title/Abstract]) OR MDX-CTLA-4[Title/Abstract]) OR Yervoy[Title/Abstract]) OR MDX-010[Title/Abstract]) OR MDX010[Title/Abstract])) OR (((((((((((((((((Programmed Cell Death 1 Receptor[MeSH Terms]) OR PD-1 Receptor[Title/Abstract]) OR PD1 Receptor[Title/Abstract]) OR Receptor, PD1[Title/Abstract]) OR Programmed Cell Death 1 Protein[Title/Abstract]) OR Programmed death receptor 1[Title/Abstract]) OR PD-1[Title/Abstract]) OR Programmed death ligand 1[Title/Abstract]) OR PD-L1[Title/Abstract]) OR Programmed Cell Death 1 Receptor[Title/Abstract]) OR anti-PDL1[Title/Abstract]) OR CTLA-4 Antigen[MeSH Terms]) OR CTLA-4[Title/Abstract]) OR CTLA4[Title/Abstract]) OR CD152[Title/Abstract]) OR Cytotoxic TLymphocyte Associated Antigen 4[Title/Abstract]) OR Cytotoxic T-Lymphocyte Antigen 4[Title/Abstract])) |
| **#3**  **Neoadjuvant therapy** | ((((("Neoadjuvant Therapy"[Mesh]) OR (Neoadjuvant Therap*[Title/Abstract])) OR (Therap*, Neoadjuvant[Title/Abstract])) OR (Neoadjuvant Treatment*[Title/Abstract])) OR (Treatment*, Neoadjuvant[Title/Abstract])) OR (Neoadjuvant immunotherapy[Title/Abstract]) |

**Table S2 Assessment of the quality of randomized controlled trials according to Cochrane Risk of Bias Tool for Randomized Controlled Trials.**

| **Study** | **Random sequence generation** | **Allocation concealment** | **Blinding of participants and personnel** | **Blinding of outcome assessment** | **Incomplete outcome data** | **Selective reporting** | **Other bias** |
| --- | --- | --- | --- | --- | --- | --- | --- |
| **Ferrarotto, R. 2022** | Low risk of bias | High risk of bias | Unclear risk of bias | High risk of bias | Low risk of bias | Low risk of bias | Unclear risk of bias |
| **Schoenfeld, J.D.**  **2020** | Low risk of bias | Low risk of bias | Low risk of bias | Low risk of bias | Low risk of bias | Low risk of bias | Unclear risk of bias |
| **Cascone, T.**  **2022** | Low risk of bias | Low risk of bias | High risk of bias | Low risk of bias | Low risk of bias | Low risk of bias | Unclear risk of bias |
| **Kaseb, A.O.**  **2022** | Low risk of bias | Low risk of bias | Unclear risk of bias | High risk of bias | Low risk of bias | Low risk of bias | Unclear risk of bias |
| **Lee, H. S.**  **2022** | Unclear risk of bias | Unclear risk of bias | Unclear risk of bias | Low risk of bias | Low risk of bias | Low risk of bias | Unclear risk of bias |
| **Rozeman, E. A.**  **2019** | Low risk of bias | High risk of bias | High risk of bias | High risk of bias | Low risk of bias | Low risk of bias | Unclear risk of bias |
| **Cascone, T.**  **2023** | Low risk of bias | Unclear risk of bias | Unclear risk of bias | Unclear risk of bias | Low risk of bias | Low risk of bias | Unclear risk of bias |

**Table S3 Assessment of the quality of included studies according to MINORS.**

| **Study** | **Q1** | **Q2** | **Q3** | **Q4** | **Q5** | **Q6** | **Q7** | **Q8** | **Q9-12** | **Score** |
| --- | --- | --- | --- | --- | --- | --- | --- | --- | --- | --- |
| **Vos, J. L. 2021** | 2 | 2 | 2 | 2 | 1 | 2 | 1 | 0 | 5 | 17 |
| **Amaria, R.N. 2022** | 2 | 2 | 2 | 2 | 1 | 2 | 1 | 0 | / | 12 |
| **Gao, J. 2020** | 2 | 2 | 2 | 2 | 1 | 2 | 1 | 0 | / | 12 |
| **Hanna, G.J. 2022** | 2 | 2 | 2 | 2 | 2 | 2 | 1 | 0 | / | 13 |
| **Marie, P.K. 2021** | 2 | 2 | 2 | 2 | 1 | 2 | 1 | 0 | / | 12 |
| **Reijers, I.L.M. 2020** | 2 | 2 | 2 | 2 | 1 | 2 | 1 | 0 | / | 12 |
| **Reuss, J.E. 2020** | 2 | 2 | 2 | 2 | 1 | 2 | 1 | 0 | / | 12 |
| **Andre, T. 2022** | 2 | 2 | 2 | 2 | 1 | 2 | 1 | 0 | / | 12 |
| **van Dijk, N. 2020** | 2 | 2 | 2 | 2 | 1 | 2 | 2 | 0 | / | 13 |

Checklist items: 1, a stated aim of the study; 2, inclusion of consecutive patients; 3, prospective collection of data; 4, endpoints appropriate to study aim; 5, unbiased assessment of study endpoint; 6, follow-up period appropriate to the major endpoint; 7, <5% lost to follow-up; 8, adequate control group; 9, contemporary groups; 10, baseline equivalence of groups; 11, prospective calculation of study size; 12, adequate statistical analyses. Items are scored as 0 (not reported); 1 (reported but inadequate); or 2 (reported and adequate). The maximum possible score is 24 points.For non-comparative studies, an overall score > 12 = high; 8–12 = intermediate; < 8 = low. For comparative studies, > 18 = high; 12–18 = intermediate; < 12 = low.

|  |  | **RCTs**  **Incidence(%,95%CI)** | **Single arms**  **Incidence(%,95%CI)** |
| --- | --- | --- | --- |
| **Any Grade** | Rash | 57.7(38.6-75.8) | 32.0(12.9-54.9) |
|  | Fatigue | 42.4(25.0-60.8) | 30.4(12.9-51.6) |
|  | Diarrhea | 26.9(15.3-40.5) | 19.8(12.6-28.2) |
|  | AST increased | 33.2(21.9-45.7) | 16.0(5.1-31.4) |
|  | Hyperthyroidism | 17.9(6.1-34.0) | 21.8(15.6-28.6) |
|  | ALT increased | 31.1(19.5-44.0) | 14.9(5.5-27.9) |
|  | Hypothyroidism | 9.5(3.6-17.8) | 11.3(3.0-23.9) |
|  | Nausea | 15.3(6.6-26.7) | 11.0(2.0-25.9) |
|  | Arthralgia | 11.6(5.7-19.2) | 23.1(13.4-34.4) |
|  | Headache | 15.6(7.7-25.5) | 13.6(8.3-20.0) |
| **Grade ≥3** | ALT increased | 9.3(2.1-20.8) | 8.2(0.6-23.1) |
|  | AST increased | 10.4(2.5-22.7) | 3.0(0.1-9.6) |
|  | Colitis | 5.5(0.0-22.5) | 5.1 (2.2-9.1) |
|  | Lipase increased | 1.0(0.0-4.8) | 14.9(3.5-32.5) |
|  | Diarrhea | 5.2(1.8-10.2) | 5.5(2.6-9.6) |
|  | Rash | 6.6(2.4-12.8) | 1.4(0.0-6.4) |
|  | GGT increased | 3.8(0.0-14.3) | 3.1(0.0-12.4) |
|  | Serum amylase increased | 1.6(0.0-5.8) | 1.0(0.0-5.1) |
|  | Headache | 0.3(0.0-2.2) | 1.0(0.0-3.4) |

**Table S4 The most common irAEs in RCTs and single arm studies in nivolumab combined with ipilimumab group.**

|  |  | **RCTs**  **Incidence(%,95%CI)** | **Single arms**  **Incidence(%,95%CI)** |
| --- | --- | --- | --- |
| **Any Grade** | Fatigue | 31.9(15.5-51.1) | 29.6(8.6-56.7) |
|  | Anemia | 31.9(15.5-51.1) | 11.6(4.4-21.7) |
|  | Rash | 28.6(8.4-58.1) | 28.6(13.2-48.7) |
|  | Pruritus | - | 21.4(11.4-33.6) |
|  | AST increased | - | 11.5(3.1-24.4) |
|  | Serum amylase increased | 7.1(0.2-33.9) | 14.4(0.2-45.1) |
|  | ALT increased | - | 17.4(8.4-29.0) |
|  | Nausea | 8.0(0.8-21.5) | 13.7(5.8-24.4) |
|  | Hyperglycemia | 54.6(23.4-83.3) | 3.6(0.0-18.4) |
| **Grade ≥3** | AST increased | - | 5.2(0.0-23.5) |
|  | Lipase increased | - | 14.3(4.0-32.7) |
|  | Colitis | - | 10.7(2.3-28.2) |
|  | ALT increase | - | 7.1(0.9-23.5) |
|  | Hyperglycemia | 18.2(2.3-51.8) | - |
|  | Diarrhea | 7.1(0.2-33.9) | 1.0(0.0-8.0) |
|  | Blood bilirubin increased | - | 3.6(0.1-18.4) |
|  | Hepatic failure | 9.1(0.2-41.3) | - |
|  | Oral mucositis | - | 4.4(0.1-22.0) |

**Table S5 The most common irAEs in RCTs and single arm studies in durvalumab combined with tremelimuma group.**

**Table S6 The most common irAEs in RCTs and single arm studies in NSCLC group.**

|  |  | **RCTs**  **Incidence(%,95%CI)** | **Single arms**  **Incidence(%,95%CI)** |
| --- | --- | --- | --- |
| **Any Grade** | Fatigue | 16.5(4.9-33.1) | 22.2(2.8-60.0) |
|  | Diarrhea | 33.3(14.6-57.0) | 15.5(5.3-29.9) |
|  | Rash acneiform | 52.4(29.8-74.3) | - |
|  | Nausea | 9.3(0.0-37.4) | 11.1(0.3-48.3) |
|  | Rash | 7.1(1.4-16.8) | 44.4(13.7-78.8) |
|  | Cough | 28.6(11.3-52.2) | - |
|  | Pruritus | 9.8(2.7-20.6) | 22.2(2.8-60.0) |
|  | Arthralgia | 2.5(0.0-20.1) | 11.1(0.3-48.3) |
|  | Asthenia | 3.7(0.0-29.5) | - |
|  | Pyrexia | 7.2(1.4-16.9) | - |

**Table S7 The most common irAEs in RCTs and single arm studies in HNC group.**

|  |  | **RCTs**  **Incidence(%,95%CI)** | **Single arms**  **Incidence(%,95%CI)** |
| --- | --- | --- | --- |
| **Any Grade** | Fatigue | 18.9(0.6-53.6) | 42.9(24.5-62.8) |
|  | ALT increased | - | 25.0(10.7-44.9) |
|  | AST increased | - | 25.0(10.7-44.9) |
|  | Arthralgia | - | 14.3(4.0-32.7) |
|  | Hypothyroidism | - | 25.0(10.7-44.9) |
|  | Rash | 28.6(8.4-58.1) | - |
|  | Diarrhea | - | 17.9(6.1-36.9) |
|  | Infusion reaction | 13.3(1.7-40.5) | 10.7(2.3-28.2) |
|  | Pruritis | - | 14.3(4.0-32.7) |
|  | Nausea | 7.1(0.2-33.9) | 10.7(2.3-28.2) |

**Table S8 The most common irAEs in RCTs and single arm studies in melanoma group.**

|  |  | **RCTs**  **Incidence(%,95%CI)** | **Single arms**  **Incidence(%,95%CI)** |
| --- | --- | --- | --- |
| **Any Grade** | Fatigue | 58.2(47.6-68.3) | 35.9(4.8-76.5) |
|  | Rash | 57.7(38.6-75.8) | 35.4(5.0-75.2) |
|  | ALT increased | 31.1(19.5-44.0) | 26.3(17.9-36.1) |
|  | Hyperthyroidism | 25.1(6.7-50.1) | 24.2(16.2-33.9) |
|  | Hypothyroidism | 14.9(8.2-23.1) | 15.1(2.3-36.3) |
|  | Diarrhea | 25.2(11.0-43.0) | 7.1(0.0-49.9) |
|  | Skin hypopigmentation | 12.8(3.4-27.1) | 20.2(12.8-29.5) |
|  | Nausea | 9.8(3.3-19.2) | 10.8(0.4-32.9) |
|  | Lipase increased | 5.7(1.8-11.6) | 16.2(9.5-24.9) |

**Table S9 Surgery delay due to irAEs in RCTs.**

| **Study** | **Cancer type** | **Intervention** | **Case** | **Number** |
| --- | --- | --- | --- | --- |
| Rozeman, E. A. | Melanoma | Ipilimumab 3 mg/kg+ Nivolumab 1 mg/kg | 1 | 30 |
|  |  | Ipilimumab 1 mg/kg +Nivolumab 3 mg/kg | 0 | 30 |
|  |  | Ipilimumab 3 mg/kg +Nivolumab 3 mg/kg | 2 | 26 |
| Cascone, T. | NSCLC | Durvalumab 1500mg+Oleclumab 3000mg | 0 | 21 |
|  |  | Durvalumab1500mg+Monalizumab750mg | 0 | 20 |
| Schoenfeld, J.D. | HNC | Nivolumab 3 mg/kg+ Ipilimumab 1 mg/kg | 0 | 15 |
| Lee, H. S. | Pleural Mesothelioma | Durvalumab1500mg+Tremelimumab75mg | 0 | 11 |
| Cascone, T. | NSCLC | Nivolumab 3mg/kg+Ipilimumab 1mg/kg | 0 | 21 |
| Kaseb, A.O. | HCC | Nivolumab240 mg+ Ipilimumab 1mg/kg | 0 | 14 |

**Table S10 Surgery delay due to irAEs in single arm studies.**

| **Study** | **Cancer type** | **Intervention** | **Case** | **Number** |
| --- | --- | --- | --- | --- |
| van Dijk, N. | UC | Ipilimumab 3 mg/kg (day 1/22)  +nivolumab 1 mg/kg (day 22)+ nivolumab 3 mg/kg (day 43) | 1 | 24 |
| Gao, J. | UC | Durvalumab1500mg+Tremelimumab75mg | 2 | 28 |
| Reijers, I.L.M. | Melanoma | Ipilimumab1mg/kg+ Nivolumab 3mg/kg | 3 | 99 |
| Reuss, J.E. | NSCLC | Nivolumab 3 mg/kg+ Ipilimumab 1 mg/kg | 0 | 9 |
| Marie, P.K. | Colorectal Cancer | Durvalumab1500mg+Tremelimumab75mg | 0 | 23 |
| Amaria, R.N. | Melanoma | Relatlimab 160 mg+ Nivolumab 480 mg | 0 | 30 |
| Andre, T. | Gastric/GEJ adenocarcinoma | Nivolumab 240 mg+Ipilimumab 1 mg/kg | 0 | 32 |
| Hanna, G.J. | HNC | Nivolumab 240 mg+ Lirilumab 240 mg | 0 | 28 |

**Table S11 Postoperative adjuvant therapy in single-arm studies and RCTs.**

| **Study** | **Neoadjuvant therapy** | **Number^1^** | **Case^2^** | **Adjuvant therapy** |
| --- | --- | --- | --- | --- |
| Reijers, I.L.M. | Ipilimumab+ Nivolumab | 21 | 17 | -7 received adjuvant nivolumab;  -10 were treated with BRAF/MEK inhibition |
| Marie, P.K. | Durvalumab+ Tremelimumab | 17 | 17 | -16 received durvalumab;  -1 received FOLFOX due to physician decision |
| Amaria, R.N. | Relatlimab+ Nivolumab | 29 | 27 | -27 received Relatlimab+ Nivolumab |
| Andre, T. | Ipilimumab+ Nivolumab | 29 | 23 | -23 received nivolumab |
| Hanna, G.J. | Nivolumab + Lirilumab | 29 | 25 | -25 received Nivolumab + Lirilumab |
| Kaseb, A.O. | Ipilimumab+ Nivolumab | 14 | 5 | -5 received Ipilimumab+ Nivolumab |
| Ferrarotto, R. | Durvalumab+ Tremelimumab | 20 | 11 | -6 received radiotherapy;  -5 received chemoradiation |
| Cascone, T. | Ipilimumab+ Nivolumab | 21 | 8 | -2 received radiotherapy;  -6 received chemotherapy |

1 The total number of people in need of adjuvant treatment mentioned in the article;

2 Number of patients receiving adjuvant therapy.
